# Prevention and Management of CMV Infections after Liver Transplantation: Current Practice in German Transplant Centers

**DOI:** 10.3390/jcm9082352

**Published:** 2020-07-23

**Authors:** Cornelius Engelmann, Martina Sterneck, Karl Heinz Weiss, Silke Templin, Steffen Zopf, Gerald Denk, Dennis Eurich, Johann Pratschke, Johannes Weiss, Felix Braun, Martin-Walter Welker, Tim Zimmermann, Petra Knipper, Dirk Nierhoff, Thomas Lorf, Elmar Jäckel, Hans-Michael Hau, Tung Yu Tsui, Aristoteles Perrakis, Hans-Jürgen Schlitt, Kerstin Herzer, Frank Tacke

**Affiliations:** 1 Department of Hepatology and Gastroenterology, Campus Virchow-Klinikum, Charité University Medicine Berlin, 13353 Berlin, Germany; cornelius.engelmann@charite.de; 2 Institute for Liver and Digestive Health, University College London, Royal Free Campus, London NW32PF, UK; 3 Section Hepatology, Clinic for Gastroenterology and Rheumatology, University Hospital Leipzig, 04103 Leipzig, Germany; 4 Department of Medicine I, University Medical Center Hamburg-Eppendorf, 20251 Hamburg, Germany; sterneck@uke.de; 5 Department of Gastroenterology and Hepatology, University of Heidelberg, 69117 Heidelberg, Germany; KarlHeinz.Weiss@med.uni-heidelberg.de; 6 Department of General, Visceral and Transplant Surgery, University Hospital Tübingen, 72076 Tübingen, Germany; silke.templin@med.uni-tuebingen.de; 7 Department of Medicine 1, University of Erlangen-Nürnberg, 91054 Erlangen, Germany; steffen.zopf@uk-erlangen.de; 8 Medical clinic and policlinic II, Campus Grosshadern, Ludwig Maximilians University (LMU), 80333 Munich, Germany; Gerald.Denk@med.uni-muenchen.de; 9 Transplantation Center Munich, University Hospital, 81377 LMU Munich, Germany; 10 Department of Surgery Campus Charité Mitte/Campus Virchow-Klinikum, 13353 Berlin, Germany; dennis.eurich@charite.de (D.E.); johann.pratschke@charite.de (J.P.); 11 Department of Medicine II, University Hospital Würzburg, 97080 Würzburg, Germany; weiss_j1@ukw.de; 12 Department of General, Visceral, Thoracic, Transplant and Pediatric Surgery, University Medical Center Schleswig-Holstein, 24105 Kiel, Germany; Felix.Braun@uksh.de; 13 Department of Medicine I, University Hospital Frankfurt, 60590 Frankfurt, Germany; Martin-Walter.Welker@kgu.de; 14 Department of Medicine, Hepatology, University of Mainz, 55101 Mainz, Germany; innere.medizin.2@klinikum-worms.de; 15 Department of Internal Medicine I, University Hospital Bonn, 53127 Bonn, Germany; petra.knippert@ukbonn.de; 16 Department of Gastroenterology and Hepatology, University Hospital Köln, 50937 Köln, Germany; dirk.nierhoff@uk-koeln.de; 17 Clinic for General, Visceral and Pediatric Surgery, University Medical Center Goettingen, 37075 Goettingen, Germany; tlorf@med.uni-goettingen.de; 18 Department of Gastroenterology, Hepatology & Endocrinology, Hannover Medical School, 30625 Hannover, Germany; jaeckel.elmar@mh-hannover.de; 19 Department of Visceral, Transplantation, Thoracic, and Vascular Surgery, University Hospital Leipzig, 04103 Leipzig, Germany; Hans-Michael.Hau@uniklinikum-dresden.de; 20 Section of Oncological Surgery and Transplantation, Rostock University Medical Center, 18057 Rostock, Germany; t.tsui@asklepios.com; 21 Department of General, Visceral, Vascular and Transplant Surgery, University Hospital Magdeburg, 39120 Magdeburg, Germany; aristotelis.perrakis@med.ovgu.de; 22 Department of Surgery, University of Regensburg, 93053 Regensburg, Germany; Hans.schlitt@ukr.de; 23 Knappschafts-Hospital Bad Neuenahr, Deutsche Rentenversicherung, Knappschaft-Bahn-See, 53474 Bad Neuenahr-Ahrweiler, Germany; 24 Department of Medicine III, University Hospital Aachen, 52074 Aachen, Germany

**Keywords:** OLT, ganciclovir, valganciclovir, mTOR inhibitor

## Abstract

Human cytomegalovirus (CMV) remains a major cause of mortality and morbidity in human liver transplant recipients. Anti-CMV therapeutics can be used to prevent or treat CMV in liver transplant recipients, but their toxicity needs to be balanced against the benefits. The choice of prevention strategy (prophylaxis or preemptive treatment) depends on the donor/recipient sero-status but may vary between institutions. We conducted a series of consultations and roundtable discussions with German liver transplant center representatives. Based on 20 out of 22 centers, we herein summarize the current approaches to CMV prevention and treatment in the context of liver transplantation in Germany. In 90% of centers, transient prophylaxis with ganciclovir or valganciclovir was standard of care in high-risk (donor CMV positive, recipient CMV naive) settings, while preemptive therapy (based on CMV viremia detected during (bi) weekly PCR testing for circulating CMV-DNA) was preferred in moderate- and low-risk settings. Duration of prophylaxis or intense surveillance was 3–6 months. In the case of CMV infection, immunosuppression was adapted. In most centers, antiviral treatment was initiated based on PCR results (median threshold value of 1000 copies/mL) with or without symptoms. Therefore, German transplant centers report similar approaches to the prevention and management of CMV infection in liver transplantation.

## 1. Introduction

Human cytomegalovirus (CMV) is nearly ubiquitous in the general population, reaching 60–70% prevalence or higher in adults depending upon ethnicity and region. CMV is also the most common opportunistic infectious complication after liver transplantation. In immunocompetent hosts, primary infections are non-serious and self-limiting but commonly lead to life-long latent infections, which presupposes continuous immune surveillance to control the virus [1,2,3,4].

Allogeneic liver transplant recipients receive immune suppression to prevent graft rejection. The degree of immune suppression must be balanced against drug toxicity and hosts’ capacity to eliminate pathogens and to control infections. Immunosuppressive therapy after solid organ transplantation increases the risk for CMV de novo infections or reactivations, leading to significant morbidity and mortality, and reduced recipient survival [5]. Reactivation may present as (i) asymptomatic CMV viremia, or as CMV disease, which may occur as (ii) CMV syndrome characterized by fever, leucopenia, neutropenia, or thrombocytopenia, or as (iii) end-organ CMV disease affecting the graft or extrahepatic organs (typically gastrointestinal tract) [4]. In addition to its direct effects, active CMV infection may cause indirect complications by increasing the risk of other opportunistic infections, initiating graft inflammation and fibrosis, elevating circulating graft antigen levels, and thus increasing the likelihood of acute or chronic rejection [5].

The risk of CMV infections is highest within the first three months after liver transplantation when the immunosuppressive therapy is kept at high intensity, requiring preventive strategies in order to avoid the development of CMV-related complications [2].

Although the clinical relevance of CMV infections after liver transplantation and the main risk factors are undisputed, there remains ambiguity among liver transplant centers regarding the optimal preventive and therapeutic measures. Therefore, we undertook a series of consultations and roundtable meetings with the leading executive physicians of German liver transplant centers to understand current practice. In this report, we summarize the current strategies for prevention and management of CMV infection after liver transplantation in Germany.

## 2. Material and Methods

A total of 22 German liver transplant centers were invited to participate in the study by completing a brief questionnaire about current institutional standards of care for CMV prevention and treatment in liver transplant recipients. Data were collected in the form of questionnaire responses from 20 centers as well as within a series of roundtable meetings and expert interviews. The data included the strategy for local CMV prevention and monitoring, diagnostic criteria, and treatment strategies in cases of CMV disease, as well as standard approaches to immunosuppression in the context of CMV. Responses were summarized and reviewed in the context of the current scientific literature.

## 3. Results and Discussion

### 3.1. Diagnostic Means for CMV

CMV’s latency after primary infections mostly in hematopoietic cells places high demands on diagnostic approaches. Strategies generally aim at identifying patients at high risk of developing clinically relevant complications, such as CMV disease. CMV serology in donors and recipients, as a marker for previous contact with the virus, is only relevant before transplantation as it determines the individual risk for CMV reactivations under immunosuppressive therapy.

CMV persists in leukocytes and immunostaining of cells for the lower matrix protein pp65, a marker for active viral replication [6], and counting of pp65 positive cells correlated well with the clinical presentation of CMV disease [7,8]. Other qualitative molecular tests, such as late pp67-related CMV mRNA (NASBA), were less sensitive than pp65-based approaches [9]. Meanwhile, the detection of the viral genome via polymerase chain reaction (PCR) replaced antigen detection techniques [10,11]. However, there is uncertainty about thresholds and indication for CMV treatment initiation as the test quality varies in terms of sensitivity and reliability [12,13]. In vitro assays based on the isolation and amplification of virus particles are of particular interest for genotyping and genome sequencing, if resistance to therapy is suspected [14]. The presence of CMV-specific T-cells can be evaluated by using CMV peptides or proteins to simulate T-lymphocytes to produce interferon-gamma (IFNγ), which can be quantified. This approach is less standardized and only available in specialized centers [15]

#### 3.1.1. Center Results for Diagnostic Approaches

PCR for CMV-DNA in blood was reported as the standard detection technique by all centers in Germany, in some cases in conjunction with pp65 antigen detection. Regular monitoring 1–2 times weekly was generally more frequent during the first 3 months post transplantation, decreasing to every 2–4 weeks or even every 3 months thereafter. Observation was maintained from up to 3 months to over 1 year depending upon the center. In cases of CMV detection, treatment was initiated at varying viral load thresholds ranging from either “positive” or 100 copies/mL up to 10,000 copies/mL; the median threshold value was 1000 copies/mL (Table 1 and Table 2).

#### 3.1.2. Diagnostic Management of CMV

Viral load quantification by real-time PCR is essential for surveillance during preemptive treatment strategies and assessment of CMV therapy response [16]. CMV DNA can be measured either in whole blood, plasma, and other specimens, such as urine or biopsies (tissue). Measuring CMV DNA from whole blood is more sensitive and allows the detection of viremia at an earlier time point compared to plasma. This is of particular value in patients with low levels of CMV DNA, in which a high test sensitivity increases the likelihood of early identification of CMV-positive patients, but might also result in prolonged treatment intervals if the aim is to achieve DNA negativity [17].

Generally, higher viral load correlates with increased risk of developing CMV disease. However, there is variability regarding the absolute threshold of the CMV load, and concentrations between 80 and 1000 copies have been proposed as indication for treatment initiation in asymptomatic patients, although the positive predictive value to develop CMV disease in low-level viremia is poor [18,19]. Higher thresholds of about 3000 genomes/mL were not able to improve the diagnostic accuracy [20]. The development of CMV DNA quantification between two time points was considered as the most reliable to predict the development of CMV-related complications [21,22,23]. The frequency of testing and interval between two tests determines its accuracy, which is essential for preemptive treatment approaches [24]. An increase of the CMV viral load by the factor 3 (≥0.5 log10 copies/mL) within one week has been proposed as an indication for treatment initiation [25]. After multiple test calibration by the World Health Organisation, international standards have been introduced, which include that test results should be reported as IU/mL to flatten the variability among assays [26].

Negative PCR blood results do not exclude tissue-invasive CMV disease, especially in conjunction with typical symptoms [27,28,29]. Diagnosis relies on PCR-based CMV detection in the tissue and might be combined with immunohistochemical techniques, most importantly targeting pp65 as antigen.

Persistent (>6 weeks) CMV DNA detection during therapy is suspicious for antiviral drug resistance and should entail resistance testing [4,30]. Relevant genes encode for UL97-phosphokinase (pUL97) and UL54 DNA polymerase. Resistance against ganciclovir usually occurs in both genes and for cidovovir and foscarnet in the UL54 gene only. Viral genome sequencing provides reliable evidence for common mutations [31], such as in the pUL97 and UL54 DNA polymerase [30,32], especially if the viral load exceeds 1000 IU/mL for pUL97 and 5000–10,000 IU/mL for UL54 [14]. Sensitivity to antiviral agents can be determined in vitro after isolation and culturing of the virus, which might be the technique of choice, especially for patients with low levels of circulating DNA [14]. However, this approach is time consuming and the long culturing period may also favor the selection of resistant viral subsets [33].

#### 3.1.3. Conclusion CMV Diagnostic Strategies

Based on a panel discussion and the current scientific literature, the following concise suggestions were generated by the authors to harmonize diagnostic approaches for CMV infections after liver transplantation:

(a) A weekly PCR-based monitoring is necessary in case a preemptive treatment strategy was chosen. Although there is no study comparing different intervals of CMV testing, studies evaluating preemptive approaches with frequent testing have shown non-inferiority to prophylaxis even in high-risk patients (D+/R−) [34,35,36].
(b) In patients with prophylaxis, testing can be reduced to monthly, followed by every 3 months (or in case of symptoms) for the first year. CMV viremia is rare during prophylaxis and current guidelines do not recommend regular testing [14,25]. However, occasional testing might detect those with CMV resistance preemptively.
(c) There is currently insufficient evidence for determining a specific standard PCR threshold for initiating anti-CMV treatment. Immediate treatment was proposed in patients with “high” viral load or organ manifestations with detection of CMV in the tissue or, in the case of “lower” viral loads, treatment should be dependent upon a second positive test result considering also its dynamic change (fold increase) or the individual risk of the patient [14].

### 3.2. Preventive Strategies for CMV Infections

Prophylactic and preemptive antiviral treatment are the two major strategies to prevent CMV disease, most importantly during the first month after liver transplantation when immunosuppression and the risk of developing CMV disease are high. The choice of strategy is based upon a risk–benefit consideration, balancing drug-associated toxicities with the risk of developing CMV disease and its complications. The latter is determined by well-known factors, such as the patients’ sero-status (donor (D)/recipient (R)) and the intensity of immunosuppression, which is high in HIV infection, and patients after re-transplantation or previous rejections [1,2,3]. Naïve recipients (R-) without acquired immunity are at the greatest risk if transplanted with an anti-CMV IgG-positive (D+) organ. Constellations with seropositive recipients (D+/R+ or D−/R+) are considered moderate risk, and D-/R- retain a low overall risk as CMV positivity only occurs through de novo infections [4]. International practice guidelines for liver transplantation are only precise in the high-risk scenario (D+/R−), recommending pharmacological CMV prophylaxis for at least 3 months [5,25,37]. However, a recent study showed that an adequate monitoring and low threshold for treatment initiation, i.e., immediately upon detection of viremia, allows the application of a preemptive therapy approach also for high-risk patients [36]. The efficacy of preemptive therapy strongly correlates with a high frequency of DNA testing in order to detect the dynamics of DNA quantification before the patient becomes symptomatic. DNA quantification thresholds for treatment initiation are less well-defined, hampering a comparison of different study results [38]. Further disadvantages are the logistic requirements to provide access to weekly CMV DNA testing and the delay between testing to receiving results, which might lead to a progression from simple ‘DNAemia’ to CMV disease [24]. Universal prophylaxis is easy to handle and does not require a strict testing regime, which might be more cost effective [4,39]. CMV prophylaxis with (val)ganciclovir also prevents other herpes virus reactivations, such as Herpes simplex virus (HSV), Epstein-barr virus (EBV), or Varicella zoster virus (VZV). However, it bares the risk of drug-related complications due to prolonged drug exposure, CMV resistance, and most importantly the risk of late-onset CMV reactivations [40,41,42,43]. Extending the duration of prophylaxis or combining both approaches, which is called the “surveillance after prophylaxis” strategy, might be adequate to avoid reactivations leading to CMV disease after treatment cessation [44,45]. Therefore, more clarity about preventive CMV management in different scenarios early after transplantation is required.

#### 3.2.1. Center Results for Preventive Strategies

In 90% of participating centers, transient prophylaxis was standard of care for high-risk D+/R-recipients (see Table 1) whilst two centers applied both strategies based on individual risk profiles. Preemptive therapy is often the preferred mode of prevention for moderate- or low-risk settings in European centers [3], which also corresponds to recommendations from German guidelines for virus infections after transplantation [14]. This was used as a standard strategy for moderate-risk R+ recipients in only 50% of German centers whilst 35% were using prophylaxis in this constellation (15% no information provided). For low-risk D−/R− recipients, in 40% of centers, preemptive treatment was the first-line approach, with prophylaxis only in one out of 20 centers (Table 1).

Almost all centers used oral valganciclovir (VGCV) (17 of 19 centers), generally at 900 mg/day (in patients with GFR > 60 mL/min), whereas ganciclovir (GCV) IV and anti-CMV IgG was the agent of choice for CMV prophylaxis only in one center each (Table 1). Oral VGCV was preferred for the reasons of logistics and the practicability of the oral versus intravenous administration route. The duration of prophylaxis was generally 3 months/100 days, though over 40% of centers reported a continuation of prophylaxis for 6 months/200 days in D+ recipients (Table 1).

#### 3.2.2. Universal Prophylaxis to Prevent CMV Disease

At present, only (V) GCV is routinely used for CMV prophylaxis in liver transplant recipients. Intravenous ganciclovir 5 mg/kg body weight per day may be given immediately after transplantation or in patients with reduced gastrointestinal absorption. In most cases, however, comparable exposure (AUC) is achieved with 900 mg/day oral VGCV [46], but GFR-dependent dose adaptation may be required. While the prodrug VGCV appears to be comparable with GCV and is approved for CMV prophylaxis in the general solid organ transplant (SOT) population [27], in some studies with liver transplants, it showed increased toxicity and lower efficacy vs. GCV [47]. In keeping with the results of Paya et al. [47], a meta-analysis of 5 trials by Kalil et al. [48] showed a 2-fold increase in the incidence of CMV disease and a 1.9-fold increase in leucopenia if patients were treated with VGCV compared with GCV. This may be due to reduced liver esterase activity in this population, and therefore lower levels of active GCV [2]. However, other studies suggest that equal efficacy [49,50] practicability reasons due to the oral formulation might explain why VGCV is still preferred for long-term prophylaxis over GCV, which requires intravenous injections.

At the recommended dose of 5 mg/kg/day i.v. for GCV and 1 × 900mg/day orally for VGCV, both drugs can be associated with hematological toxicities, which typically limit the duration of CMV prophylaxis or require dose adjustment [14].

Late-onset disease after prophylaxis occurs most frequently in D+/R− recipients [39]. While (V) GCV is effective at reducing the incidence of viremia from between 36% and 100% down to <5% [39,43,51], late-onset disease appears to reduce much of the anticipated advantage of “universal prophylaxis”, with up to a 25% incidence after prophylaxis vs. 8.3% in the 3–6-month period under preemptive treatment [4].

As an additional strategy, particularly in D+/R− recipients, some studies suggest that the increased levels of late-onset disease may be mitigated by extending prophylaxis from 100 up to 200 days. Data is limited in liver transplant recipients, but results from a study in kidney recipients showed a substantially greater incidence of late-onset disease with 38.7% of patients receiving 100 days of prophylaxis vs. 21.3% (*p* < 0.001) of patients after 200 days of prophylaxis post transplantation [52]. CMV-specific immunoglobulin might be an alternative to (V) GCV as it is also approved for prophylactic use in asymptomatic patients [14].

Surveillance after prophylaxis might be an alternative in spite of prolonged drug exposure. However, studies published so far are generally lacking an adequate design to show a true benefit and, moreover, maintaining a weekly DNA testing rate even beyond 6 months after transplant is difficult to implement. Therefore, surveillance after prophylaxis may be restricted to those at high risk of late-onset CMV disease [45].

#### 3.2.3. Preemptive Treatment to Prevent CMV Disease

Preemptive treatment aims at starting antiviral therapy as soon as CMV replication is detected or triggered by a predefined threshold of viral replication so that the development of CMV-related diseases or complications is preempted. Consequently, this strategy uses intensive monitoring of viral load, typically weekly, using nucleic acid testing (PCR) for at least 3 months to detect potential increases in viral replication. This intense monitoring of CMV by PCR allows the immediate initiation of suppressive treatment before symptoms emerge but does not avoid viremia [3,39]. Less frequent DNA testing results in higher rates of CMV disease, negatively impacting on graft survival, and thus making it less effective compared to prophylaxis [53,54].

Most commonly, preemptive treatment is initiated upon reaching a threshold viral load or dynamic increase rather than based on the simple virus detection [2,25]. Expectedly, viremia rates are significantly higher during preemptive treatment than during prophylaxis but that does not translate into an inferior outcome, which basically proves the clinical efficacy of a preventive approach.

In a relatively small study, Onor et al. reported significantly higher rates of CMV viremia rates during preemptive treatment at 3 months (4.9% prophylaxis vs. 50.0% preemptive, *p* < 0.001), but secondary efficacy endpoints including the incidence of CMV tissue-invasive disease and acute cellular rejections, were not different between both preventive strategies [55]. Other studies have shown success with preemptive treatment even in D+/R− patients. Sun et al. demonstrated low rates of CMV disease (<2% overall) although having one third D+/R− patients in their cohort [39] and Lautenschlager et al. reported that most reactivations under the preemptive strategy in R+ recipients were low level, responded to treatment, and did not lead to death or loss of the graft [34].

#### 3.2.4. Conclusion CMV Prevention Strategies

Suggestions for strategies of how to prevent CMV infections after liver transplantation were generated based on a panel discussion among all participating centers and interpretation of the current scientific literature:

- Transient prophylaxis in high-risk patients (typically VGCV 900 mg/day if renal function is normal) should be given for at least 3 months and may be extended to 6 months, if no toxicities are observed, and can be performed for 3 months in intermediate-risk patients. Extension of the treatment duration to 6 months relates to a study in kidney transplant recipients, where 200 days in high-risk patients reduced the risk of CMV disease over 100 days of prophylaxis [56]. Alternatively, preemptive management seemed to be equally efficacious [36].
- The choice of strategy for preventing CMV disease and associated complications is at the discretion of the center and should also consider patients’ individual risk and logistic capacities, in particular in the outpatient setting.
- If weekly PCR-based monitoring during preemptive treatment strategy cannot be provided for patients with high and intermediate risk, a switch to prophylaxis is preferred. Longer testing intervals might increase the risk of undetected viremia and thus increased risk of CMV disease [38].

### 3.3. Therapy of CMV Infections and Disease

Manifest CMV diseases require adequate therapy, as suboptimal dosing bears the risk of treatment failure or the development of resistance [24]. Oral VGCV and intravenous GCV are associated with similar outcomes after CMV disease and thus both may be used as treatment [57]. However, intravenous GCV should be preferred if the disease is life threatening, and when optimal drug availability is essential.

#### 3.3.1. Center Results for CMV Treatment

German liver transplant centers almost universally followed the standard treatment dose for (V)GCV in SOT recipients of VGCV 2 × 900 mg/day or GCV 2 × 5 mg/kg/day IV [25]; only 3 of 16 responses reported 900 mg/day or equivalent. GCV was favored for the initial treatment of symptomatic cases, with VGCV being used thereafter. Of the 12 centers that detailed treatment durations for CMV reactivation, most centers (75%) continued treatment for 1–4 weeks after testing negative. Three centers treated for 3 months. Six of the reporting centers stated that treatment was followed by 3–6 months of prophylaxis. In two centers, supplementary anti-CMV IgG was also used (Table 1 and Table 2).

#### 3.3.2. Treatment of Manifest CMV Infections

(V)GCV, foscarnet, and cidofovir, which target the viral DNA polymerase, are currently used to treat CMV disease; however, they carry a high potential for toxicity. Alternative anti-CMV drugs, such as letermovir, are still lacking robust data in liver transplant patients [58].

GCV and its orally bioavailable ester prodrug VGCV are analogues of deoxyguanosine [46,59]. Following intracellular activation by viral thymidine kinase (UL97), GCV blocks viral replication by inhibition of the viral DNA polymerase (UL54). GCV has to be given IV, while the newer prodrug VGCV has an excellent oral bioavailability (ca. 60%) (46). Since GCVs’ clearance is almost entirely renal, dosing requires adjustment per GFR [60]. GCV is also known to be toxic for bone marrow progenitor cells in approximately the same concentration range as its anti-CMV activity (CC_50_ bone marrow: 2.7 ± 0.5 µM, EC_50_ CMV: 1.7–5.9 µM) [60]. Of particular relevance in this population, the use of GCV can cause leucopenia and neutropenia as well as thrombocytopenia and anemia, in addition to its renal and genetic toxicities [59].

Foscarnet is a pyrophosphate inhibiting the viral polymerase. Since Foscarnet does not require activation by UL97, it is commonly used as a second-line IV treatment for UL-97-mediated GCV resistance. UL54 mutations may nonetheless cause cross-resistance [61]. Of particular note, treatment with Foscarnet is associated with electrolyte disturbances and nephrotoxicity, such that combination with other nephrotoxic drugs, including tacrolimus or cyclosporine, should be avoided [61] and, moreover, its use in liver transplant recipients is off-label.

Cidofovir is a cytidine analogue targeting the viral UL54 DNA polymerase. Cidofovir is activated by intracellular kinases, i.e., independently of UL97, and so also remains active against these escape mutations. However, due to its profound toxicity (renal, myelo-, and genetic [61,62]), Cidofovir is generally restricted to third-line treatment. Cidofovir must be given alongside probenecid to minimize its severe renal toxicity, and its use in liver transplant recipients is off-label. As with Foscarnet, co-administration of Cidofovir with other nephrotoxic drugs is contraindicated.

A recently introduced new treatment approach is the terminase inhibitor letermovir, which may be an effective prophylaxis in this population and does not cause myelosuppression. However, it is currently only approved for use in stem cell recipients [58]. Letermovir targets the viral terminase complex, and is therefore not subject to cross-resistance via UL97 or UL54 mutations. Despite its effective use in a phase II study in kidney transplant recipients [63] and a case report of successful off-label use in a GCV-resistant heart transplant recipient [64], there remains insufficient evidence for a recommendation in liver or other SOT recipients at this time. Caveats include its narrow spectrum of activity (it is specific to CMV) and early case reports of essentially absolute resistance developing at therapeutic doses in vivo [65]. Nevertheless, letermovir may emerge as an effective alternative for treatment or prophylaxis, especially considering the lack of side-effects, notably its lack of myelotoxicity [58], and minimal drug–drug interactions.

Treatment duration still remains at each center’s discretion. As premature treatment cessation substantially increases the risk of CMV resistance [66], treatment should be continued until CMV eradication, e.g., until a lack of CMV detection on two independent sampling dates at least one week apart [25]. CMV hyperimmunoglobine is not approved for the therapy of CMV infections, although some centers use it as an alternative to VGCV [14].

#### 3.3.3. Conclusion Treatment of CMV infections

Suggestions for strategies of how to treat CMV infections after liver transplantation were generated based on a panel discussion among all participating centers and interpretation of the current scientific literature:

- (V)GCV is the first-line treatment for CMV disease, although robust efficacy data for liver transplant patients are limited. Although both agents seem to be equally effective [57], VGCV is preferred due to its oral administration.
- Foscarnet and cidofovir can be used as second-line therapy, especially after insufficient treatment response to VGCV, but its high toxicity requires closed clinical follow-up. Foscarnet is as effective as GCV but remains a second-line therapy due to nephrotoxicity [67]. Cidefovir is less well studied as it also comprises a range of potential side-effects [14].
- Since much of the risk of CMV infection is related to the myelosuppressive effects of (V)GCV, the use alternative therapies, such as letermovir, should also be considered, at least as a considerably safer alternative to foscarnet or cidofovir. However, evidence in solid organ transplant recipients is still sparse [63].
- In case of asymptomatic viremia, VGCV at 2 × 900 mg/day until 2 consecutive negative PCR results with a time interval of one week are obtained. In cases of symptomatic disease, such as pneumonia, colitis, hepatitis, or CMV syndrome, a 2-week therapy with GCV (or longer, if no undetectable CMV levels are achieved) followed by oral VGCV is recommended. Additional use of CMV-specific IgG may be considered. If CMV viremia persists for more than two weeks despite (V)GCV, foscarnet should be initiated as a second-line treatment and resistance testing should be performed (14).

### 3.4. Immunosuppressive Therapy and CMV Infections

In liver transplant recipients, calcineurin inhibitors (CNIs, tacrolimus or cyclosporine) form the core of immunosuppressive therapy; however, they are associated with renal and neurotoxicity as well as an increased risk of developing malignancies. CNI minimization by combination with anti-metabolites (mycophenolate, MMF) and steroids is the current strategy to avoid CNI-related side-effects. Contrary and less beneficial effects are known with respect to CMV, as MMF increases the severity of CMV infections [68]. The use of mammalian target of rapamycin (mTOR) inhibitors has emerged as an alternative to MMF in CNI-minimizing approaches or as a backbone for CNI-free therapeutic regimes [69]. Everolimus, an mTOR inhibitor, downregulates cap-dependent translation from messenger RNA into proteins, which is triggered through activation of the phosphatidylinositol 3-kinase-Akt-mTOR pathway. As CMV uses the same pathways for replication, everolimus has additional virostatic effects on CMV at early stages of infection [69,70]. Therefore, the choice of immunosuppressive agents might be an adjunctive to antiviral strategies to modify the development of CMV-related complications.

#### 3.4.1. Center Results for Immunosuppression

Immunosuppression in liver transplant recipients has been extensively and recently reviewed [69,71], also in the context of current German practice [69], but not specifically in relation to CMV prevention and recurrence. All 20 centers provided information on current de novo immunosuppression strategies. Of these, 10 centers use induction therapy (basiliximab) as a standard option.

Similarly, all centers reported using a CNI as a standard de novo immunosuppressant for liver recipients, usually tacrolimus. This was generally combined with MMF (in 16/20 centers) and/or steroids (16/20). In about half of the centers, transition to an mTOR inhibitor (i.e., everolimus) was standard practice either after wound healing or for tumor, renal impairment, or patients with higher CMV risk.

In cases of CMV viremia or disease, most (14/20) centers reported reducing or stopping MMF and initiating mTOR therapy.

#### 3.4.2. mTOR Inhibitor-based Immunosuppressive Strategies to Reduce the Risk of CMV Disease

There are several large meta-analyses showing that the use of mTOR inhibitors reduces CMV replication by 2- to > 3-fold vs. MMF in various types of solid organ transplantation [70,72,73,74], and a switch from MMF to mTOR inhibitor as an additive therapy to tacrolimus improves the CMV infection-free survival [75]. Similarly, mTOR inhibitor-based immunosuppression reduces viral replication by 2.3- to 2.5-fold [76] and mTOR-containing immunosuppressive regimes reduced the incidence of CMV infection in kidney transplant patients in comparison to CNI-based immunosuppression [77]. This anti-CMV effect of mTOR inhibitors was stronger with earlier and de novo use [78]. However, evidence for liver transplant recipients is sparse, and while mTOR inhibitors markedly reduce CMV-related complications, they are also associated with anemia and leucopenia/neutropenia, favoring their use in combination with a preemptive CMV treatment approach as a preventive strategy [78].

#### 3.4.3. Conclusion Immunosuppression and CMV Disease

After discussion of the current literature within the review panel, the following conclusions were drawn by the authors regarding changes in immunosuppressive treatment upon CMV infections:

- In patients with CMV infection, cessation of MMF is recommended and tacrolimus trough levels should be kept at a low level with 5–7 µg/L if applicable.
- After repetitive CMV infection, an mTOR inhibitor-based strategy either in a CNI-minimizing manner or as the immunosuppressive backbone can be considered unless contraindicated. These suggestions are based on the fact that higher immunosuppression with mycophenolate derivates or CNI have a boosting effect on viral replication whereas the mTOR inhibitor acts as virustatic [77]. Therefore, the evidence suggests a switch to an mTOR inhibitor-based strategy as an adjunct to conventional approaches.
- However, immunosuppressive therapy remains an individual treatment, which must be tailored according to individual patient needs and characteristics.

## 4. Summary

CMV disease is the most frequent infectious complication after liver transplantation, and a structured management, including prevention, early detection, and adequate treatment, is required to avoid negative effects on patient and graft survival. We herein provided a consensus-type summary of the current practice at German liver transplant centers, roundtable discussions with experts, and a review of the current literature. It is, however, important to emphasize that future studies should assess the efficacy of different (center-specific) approaches to CMV diagnosis and treatment in a prospective setting. At present, PCR-based CMV DNA detection is the diagnostic gold standard for measuring the blood/plasma viral load and might be complemented by pp65 staining, in situ hybridization, or PCR in tissue samples in symptomatic patients with suspected tissue-invasive CMV infection. Universal CMV prophylaxis with VGCV should be considered 3-6 months after transplantation in patients at high risk (D+/R−) and 3 months in patients with moderate risk (R+). Symptomatic patients with CMV disease and a high risk of complications are commonly treated with intravenous GCV whilst asymptomatic patients receive oral VGCV. Immune suppression with mTOR inhibitors can be an adjunctive measure as part of the management of CMV infections.

## Figures and Tables

**Table 1:** Summary of prevention strategies as reported by German liver transplant centers.

Seroprofile	Centers Reporting Standard Prevention, n/N (%)		Centers with Prophylaxis Drug, n/N (%)		Centers with (V)GCV Prophylaxis dose, n/N (%)		Centers with Prophylaxis Duration, n/N (%)	
	Preemptive	Prophylaxis	(V)GCV	Other	450 mg/d	900 mg/d	3 Months (or 100d)	6 Months (or 200d)
D+/R−	3/20 (15%)	19/20 (90%)	18/19 (95%) ^a^	1/19 (IgG) (5%)	2/18 (11%) ^b^	15/18 (83%) ^b^	9/17 (53%) ^b,c^	7/17 (41%) ^b,c^
D+/R+	10/20 (50%)	7/20 (35%)	7/7 (100%)	-	1/7 (14%)	6/7 (86%)	4/7 (57%)	3/7 (43%)
D−/R+	10/20 (50%)	7/20 (35%)	7/7 (100%)	-	1/7 (14%)	6/7 (86%)	6/7 (86%)	1/7 (14%)
D−/R-	8/20 (40%)	1/20 (5%)	1/1 (100%)	-	0/1 (0%)	1/1 (100%	1/1 (100%)	0/1 (0%)

**^a^** included 1 center reporting intravenous GCV 2x5mg/kg/day for initiation, followed by VGCV 900 mg/day for maintenance **^b^** 1 center did not provide details **^c^** 1/17 center reported 2 months standard prophylaxis in D+/R− recipients. VGCV—valganciclovir, GCV—ganciclovir.

**Table 2:** Current approaches in German transplant centers to immune suppression and human cytomegalovirus (CMV) treatment in liver transplant recipients with CMV disease.

Use of Induction Therapy	11/20 (55%)
Standard de novo IS	
CNI + CS	4/20 (20%)
CNI + MMF	3/20 (15%)
CNI + MMF + CS	12/20 (60%)
CNI + MMF/mTOR	1/20 (5%)
IS adjustment with CMV	
Stop/pause MMF	15/16 (94%)
Reduce/stop CNI	2/16 (13%)
Reduce/stop CS	2/16 (13%)
Introduce mTOR (±CNI)	9/16 (56%)
Viral load threshold for treatment (copies/mL)	Median = 1000 copies/mL
Any positive value	4/17
≤200	2/17
>200 to ≤1000	8/17
>1000	3/17
Treatment	
(V)GCV	16/16
Organ therapy and IgG	1/16 (6%) (+1/16 if leucopenic/renal insuff.)
Dose	
Viremia: N = 6	
VGCV: 900 mg/day PO	2/6 (33%)
VGCV: 1800 mg/day PO	4/6 (67%)
Symptomatic: N = 6	
GCV: 2 × 5 mg/kg/day	5/6 (83%)
VGCV: 1800 mg/day	1/6 (17%)
Not specific: N = 9	
GCV: 2 × 5 mg/kg/day	7/9 (78%)
VGCV: 450 mg/day	1/9 (11%)
VGCV: 900 mg/day	3/9 (33%)
VGCV: 1800 mg/day	2/9 (22%)
Duration, N = 12	
Up to ≤ 2 weeks negative test	7/12 (58%)
Up to > 2–4 weeks negative test	2/12 (17%)
2 weeks treatment	1/12 (8%)
3 months treatment	2/12 (17%)
Followed by prophylaxis	6 ^a^

^a^ No denominator possible since this information was not requested but provided spontaneously by some centers. VGCV—valganciclovir, GCV—ganciclovir, CNI—calcineurin inhibitor, CS—corticosteroid, MMF—mycophenolat mofetil, mTOR—mTOR inhibitor, CMV—human cytomegalovirus.
